# Mr. Pearson's Life of Mr. Hey

**Published:** 1824-03-01

**Authors:** 


*The Life of William Hey, Esq. F.R.S. &c.* By John Pearson, F.R.S. &c. In Three Volumes, 8vo. Second Edition. London, 1823.

We believe our greatest enemies will not accuse us of advocating the cause of irreligion or immorality; and yet, we fear that, from the notice which we shall take of the work before us, the pious biographer of the very pious Hey, will be induced to class us rather among the sinners than the saints. We apprehend, however, that, in shunning impiety, it is not necessary to run into fanaticism. For ourselves, we confess, that we have always thought Pope's maxim a just one, "To enjoy is to obey"—provided that enjoyment does not interfere with the happiness of others, or contravene any of the obvious laws of nature or reason. This does not appear to quadrate with the rigid morality of Mr. Hey, whose religious principles would not sanction such innocent amusements as a game of whist, or a theatrical representation! One of the objections which Mr. Hey urged against whist appears to us to be exceedingly overstrained. It is this:—

> "Card-playing, with all games of hazard, partakes of the nature of a *lot*, which is laying aside our own judgment for the time, and referring the matter to the interference of Divine Providence."

Assuredly it is difficult to entertain a very high degree of respect for the intellect of a man who could suppose the supreme ruler of the universe offended by the mere act of taking a hand at whist, or viewing in a theatre a scene of human passions and actions from the pen of a Shakespeare! We never can coincide with those devotees of solemnity, who would banish recreation from life, and render it a gloomy, or, at all events, an insipid scene of preparation for another world. Do we not see the beasts of the field, the birds of the air, and the fishes of the deep, frisk and gambol, and flutter about with exuberance of joy, whenever the immediate concerns of their existence are provided for?—And should man, whose countenance is peculiarly calculated to express the emotions of pleasure, be the *only* moping melancholy being on the face of this beauteous globe? It is in vain to tell us, that if we are merry we should sing psalms and spiritual songs. There is a time for all things—a time to work, and a time to play—a time to weep over the unavoidable miseries of humanity—and a time to enjoy the few hours of festivity and hilarity, which are permitted to mortals in this transitory and chequered sojourn upon earth.

In making these reflections, we are by no means inclined to deny that—"the biography of a man in whom superior intelligence, considerable attainments, and professional eminence, were combined with a consistent and uniform recognition of religious, moral, and social obligations, matured into habit, and operating with the force and constancy of a living principle, might be contemplated with advantage."* We would only question the probability of examples of ultra-austerity being followed. We think, there cannot be a doubt that, the rigid and over-scrupulous consciences of the methodists have often brought ridicule on true religion, and supplied many a powerful handle for infidelity to subvert its best principles. But we must now proceed to the work before us.

In a long, and able preface, Mr. Pearson has touched upon many highly interesting subjects. He acknowledges his moral obligations to the late Mr. Hey, under whose hospitable roof, he spent three of the happiest, though sickliest, years of his life; viz. the years 1777-8-9. The able biographer avers that, the example of Mr. Hey is adapted to the ordinary condition and circumstances of men in society—"to those who are actively employed in some particular vocation, yet are desirous of devoting the *intervals of business* to the fulfilment of public, domestic, and personal *duties.*" Now we appeal to Mr. Pearson, and to the reader, whether this plan does not completely exclude what is termed recreation, or amusement, from the routine of life?—and, if so, whether this example be adapted to the ordinary condition and circumstances of men in society? For our own parts, we think it is *not* adapted for men in society, as we are not inclined to give up those innocent amusements and recreations which smooth the brow of care, and relax the mind after the harrassing anxieties of business and duty. Mr. Pearson informs us, that Mr. Hey had no propensity to "a high-strained and rigorous interpretation of the maxims of morality delivered in the Holy Scriptures"—but what, we ask, is the solemn trifling about the sin of dealing a hand at whist, or witnessing a theatrical representation, but a "high-strained and rigorous interpretation of the maxims of morality?"

"True piety," says Mr. Pearson, "enjoins nothing contradictory to the constitution of human nature, as it came from the hands of the great Creator. It allows of no admixture of man's invention in what is offered to our faith, and imposed on our practice: neither does it admit of any compensation for unbelief, by a creditable attention to social and relative duties."—*Preface*, xxvi.

---

* Mr. Pearson's Preface, p. xvi.

Be it so—but who is to fix the standard of faith? No two sects, even of the Christian religion, are exactly agreed in the articles of belief—nor do we think there is one of them entirely free from "admixture of man's invention." This being the state of things, we humbly conceive that "attention to relative and social duties" does and ought to compensate for doubts as to *all* the articles of faith which are offered for our acceptance by various interpreters of Holy Writ. Belief or unbelief is not a voluntary act. We cannot force the one or the other—and surely we are not punishable for either, merely as convictions of the mind. But we may be wrong in our beliefs or unbeliefs; and it is when we endeavour to propagate our false creeds or doubts to others, that we become culpable.

In this preface Mr. Pearson takes up the subject of infidelity as particularly imputable to the medical profession. We agree with our author in thinking that the charge has been by far too general. There probably is not a greater proportion of sceptics in medicine than in the law—or even in the church; but it has, by some means, become more noticed there than elsewhere. It is not very apparent, as Mr. Pearson observes, why the study of medicine should have any direct tendency to render men irreligious and immoral beyond many other studies. We do not think that it *has* done do. On the contrary, we are firmly persuaded that there is *less* immorality in the medical, than in either of the other two learned professions—infinitely less indeed than in the law.

But whatever may be the state of religion in the medical profession, there is unquestionably, as Mr. Pearson remarks, some improvement in the conduct and manners of its members towards each other; and a still greater amelioration may be hoped for and anticipated, not only from the example of living practitioners, whose blameless integrity, humanity, and generous contempt of all mean artifices, diffuse a beneficial influence through the society of which they are members, but also from the amenity which begins to prevail in the writings of professional authors, and which promises, in a few years, to banish scurrility, altercation, and abuse entirely out of the confines of medical literature. There is still, however, a very considerable admixture of evil in *medical ethics*, a correction of which is one of the greatest desiderata in our profession. In medical science there are, unfortunately, but few principles so fixed as to command general assent, and few methods of treating diseases reduced to a matter of positive rule—in short, there is scarcely any theory which is not open to animadversion, or practice which is not obnoxious to censure, especially if it prove unsuccessful. Hence a man, disposed to detract from the merit of his professional brethren—to disparage their qualifications, or impute to them incompetency, may easily find specious pretences for arraigning their conduct, and a willing audience to listen to his malicious and interested insinuations.

The following piece of ethical morality we recommend to every class of the profession. A strict attention to it would do more to raise the character of medical men in the eyes of the public than the splendid talents of a thousand HUNTERS.

"Truth and justice forbid us to sanction the errors, or justify the mistakes, of our professional brethren; but a man may correct these with good temper and delicacy: he may forbear to disclose them further than his duty to the patient shall imperiously require; carefully abstaining from whatever would tend to ruin the reputation of another in the mind of the patient. It may, indeed, occur in our profession, as in other departments of civil life, *that a man may be so actuated by ambition, inflated with self-conceit, or intoxicated with vanity, as to lose all perception of right and wrong, of decency, good breeding, and humanity. This state of moral insensibility will be commonly proof against all remonstrance and admonition, and must be left to receive its just retribution from the detestation it will excite, and the hostility it will kindle, in all those who have the misfortune to encounter it.*" lxv.

It is scarcely possible, as Mr. Pearson remarks, for a man, even of the best disciplined mind and purest intentions, to be much engaged in professional business without suffering from the suspicions, misrepresentations, or injustice of those with whom he is concerned. Wisdom and experience will teach him the advantage of being little moved by those things. Under circumstances so irritating to the natural feelings, a silent forbearance will be commonly more discreet, as well as philosophic, than angry recrimination and passionate efforts to resent and repel such injuries.

"An elevation of mind, a magnanimity of spirit, founded on conscious rectitude, will commonly maintain a man under these vexations, and enable him to proceed in the course of his duty, without condescending to notice the assaults of his enemies. If we cannot remain ignorant of the ill offices, to which the peculiarity of our vocation may expose us, we can at least withdraw our attention, or try to forget them. By thus steadily resisting the suggestions of pride and revenge, we shall best secure our own peace of mind and promote our truest interests." lxvii.

It would be endless to enquire into the various schemes, by which men have endeavoured to raise a reputation, attract employment, or gain an ascendancy over their patients. He who possesses true greatness of mind and inflexible probity, will neither condescend to study or to practise what honorrable principles or feelings cannot approve—and after all, the surest and most successful policy will be found in competent ability, integrity, diligence, humanity, and unaffected kindness. These will stand us more in stead than all that craft, and cunning, and artifice can affect—and without these, the highest tide of prosperity will neither confer present satisfaction, nor insure our future happiness.

The work before us consists of three parts—the professional life—the moral and social life—and the biographer's remarks on the professional writings of Mr. Hey. We shall endeavour to present our readers with a sketch of each.

1. Mr. Hey was born in the village of Pudsey, near Leeds, on the 23d of August, 1736. At the age of four years he lost his right eye, by a wound with the point of a pen-knife; but he retained the faculty of vision in great perfection with the left, till a very late period of his life. During youth, Mr. Hey was remarkable for sprightliness and activity, engaging in all the sports of children with great ardour and vivacity. This shews us how much our natural disposition may be altered by education and accidental circumstances in the course of early life. Mr. Hey's parents were very pious, and inculcated, with great care, the principles of religion and morality in the mind of their child. At 14 he was placed as an apprentice with Mr. Dawson, Surgeon, at Leeds, somewhat contrary to his own inclinations, which were for a sea-life. As an apprentice he conducted himself with great industry and fidelity, submitting to many offices which ought not, and now seldom are, imposed on young men in similar situations. In his curiosity respecting the effects of opium, he swallowed such a quantity once as seriously endangered his life. In the autumn of 1757 he went to attend lectures in London, under the able teachers of that time. He was a pupil and dresser at St. George's Hospital under Mr. Bromfield—attended the medical prelections of Dr. Donald Monro—the lectures on midwifery of Dr. Mackenzie, &c. In this period a gentleman in London proposed to the young student a matrimonial alliance with his daughter. Mr. Hey referred the matter to his father, like a dutiful child, and by his advice declined the connexion. Mr. Pearson lauds the conduct of Mr. Hey upon this occasion, and recommends it as an example to young gentlemen in general. Mr. Hey's medical studies were never allowed to interrupt the exercise of his religious duties. The gentle persuasions or the scoffs and ridicule of his fellow students were equally resisted by his unbending mind. Mr. Hey's industry and proficiency, however, secured him pretty well from the attacks of his less pious companions, who were often obliged to have recourse to him for advice and assistance. "A serious young man," says he, "who followed me, did not escape so well. His fellow students at St. George's Hospital tossed him in a blanket."

"Mr. Hey was a Christian, a steadfast believer in divine revelation; and under the sure guidance of this supernatural light, he repelled the shafts of infidelity, resisted the seductions of unlawful pleasures, and exhibited an instructive example to all his young associates, of those several virtues, which are the honour and ornament of youth, and secure the approbation and friendship of the truly wise and good." 15.

Having completed his studies in London, Mr. Hey's father proposed a temporary residence in Paris for professional improvement. The young student, after weighing well the matter, declined risking his morals in that licentious metropolis. This, we think, was carrying matters rather too far. If he had had confidence in his own piety he need not have feared Paris, which indeed holds out no greater temptations, after all, than London; and his religious principles would have attracted far less attention in the former than in the latter city. It was in London that Mr. Hey undertook the very difficult task of strictly governing his thoughts, which control over his intellectual operations he maintained to the end of his life. He determined that he would meditate on a given subject while he was walking a certain distance, and that *then* he would turn his attention to some other topic. He was thus accustomed to pass through the streets of London, investigating the various subjects to which his thoughts had been directed by the lectures or other professional occupations. The effects of this habit remained with him through life—"and he found it," says his biographer, "of admirable use not only in preserving him from a swarm of impertinent ideas, but in enabling him to form a correct judgment on many points pertaining to divine and human knowledge." It is not every mind, however, that will bear such perpetual exertion; and it is questionable whether much is gained, upon the whole, by such a plan. The mind, like the body, requires rest, and we have generally found it best not to exercise *both* too much at the same time.

Mr. Hey settled at Leeds as a surgeon-apothecary, but never waved any operation, even of the most formidable kind, as lithotomy, the operation for hernia, &c. He performed lithotomy three times in the first year of his practice, and yet it was ten years before the emoluments of his profession were equal to the expenses of his family. It is very true, as Mr. Pearson remarks, that the talents and skill of a medical man cannot be known immediately on his announcing himself as a candidate for public confidence. "He must wait on the slow operation of time, and on the intervention of circumstances favourable to the disclosing of his professional abilities." It must, however, be a very unusual concurrence of adverse events which can finally obstruct persevering diligence and competent professional acquirements. Indeed we are convinced, that want of ultimate success, in such cases, rarely, if ever, depends on any other than *moral* causes, quite independent of professional. Mr. Hey himself appears to have suffered retardation in his medical career from "the austere and unbending features of his virtue." Such austerity we consider as unnecessary, at least, in the physician or surgeon, whose conduct should always exhibit benevolence, humanity, and virtue, but never savour of the devotee, and still less of the fanatic.

In 1761 Mr. Hey married Miss Alice Banks, of Craven, in Yorkshire; and during the year 62-3 had the charge of the Leeds workhouse, the only institution at that time appropriated to the relief of the sick and hurt of that town. In 1767 an infirmary was established, and Mr. Hey was appointed one of the surgeons, having taken an active part in giving origin to the institution. It is now capable of containing nearly 140 beds. In 1768 Mr. Hey and some others formed a medical society, which met once a month for the discussion of professional subjects, but did not continue long. Mr. Hey and Dr. Priestly, though so opposite in their theological tenets, were yet friends in scientific pursuits, and maintained a literary correspondence, always avoiding the subject of religion. Publicly, however, Mr. Hey endeavoured to counteract the tendency of Dr. Priestley's doctrines, by writing answers to them, one of the best of which was on the "Divinity of Christ." In 1773 Mr. Hey received an injury in his knee which caused lameness for life. By the year 1778 it appears that his reputation as an operating surgeon stood high, and people came from a considerable distance to be under his care. He had now a large family—eleven children—when he received a second injury, while mounting his horse, which, for some time, threatened his existence. He felt this afflictive dispensation as became a father and husband, but betrayed no discontent, impatience, or dejection of mind. "His religious principles were now tried, and he was enabled to sustain this visitation with humble submission, and a meek acquiescence in the divine will, relying with an unsuspecting confidence upon the gracious declarations of his heavenly father."

"A manly submission to events, which no human power nor wisdom can control, merits respect, and commands admiration: yet it must be acknowledged that the triumph of human fortitude receives an excellence and a dignity of a higher order, from being united with a temper of religious devotion, with a cheerful resignation to the appointments of infinite wisdom, and with a sincere and uniform desire that the supreme will may be fulfilled, whatever be the issue of the present dispensation." 49.

His lameness promising to be of long duration, he visited the metropolis for advice, and thence proceeded to Bath, where he remained for some time, and ultimately returned to Leeds in good health, but lame for life, being unable to walk across a room without the aid of a crutch. He now, though with much reluctance, (a very unusual thing with young medical men,) started his carriage, and had no reason to regret this additional expense, since it proved, under Divine Providence, (as Mr. Pearson expresses it,) one great means of protecting his health, enlarging his sphere of usefulness, and protracting a valuable life.*

Mr. Hey united the practice of midwifery to that of surgery, and wrote an Essay on Puerperal Convulsions, but did not publish it, lest it should lead him into a controversy with Dr. Denman, whose opinions on this subject were at variance with his own. In 1812 Mr. H. resigned his office of surgeon to the infirmary, being then in his 77th year, and after having held the appointment with great honour to himself and advantage to the public for a space of more than 45 years! The general board of trustees paid Mr. H. many well-deserved compliments on the occasion, and immediately elected his son to the situation vacated by the father. He still took an interest in the infirmary, and effectually contributed to change a measure in the institution which had long been acted on to its detriment. This was the election of the house apothecary, at a low salary, by the trustees at large, who, of course, are very incompetent, in general, to judge of the merits or qualifications of such a person. The law was reversed, and the election was vested in the medical officers of the charity. It

---

* The question is often agitated whether a young medical man should set up a vehicle with the view of *getting* practice by making a more respectable appearance? We think there can be little doubt that in large towns, and especially the metropolis of a country, an equipage has some effect in the way above alluded to. But we cannot believe that it is consonant with either policy, good sense, or common honesty, to start a carriage with the mere view of acquiring practice, and before the practice has risen to the point which justifies the expense and secures against the chance of being obliged to put down the equipage, should the adventure (for so it ought to be called) fail to effect the purpose designed. This dangerous experiment, however, is not unfrequently tried, and sometimes succeeds. We do not recommend it to our junior brethren.

would probably be better for institutions, if all the medical officers were elected in this manner—or rather if the election was vested in the profession generally, every member of which should have a vote.

"The election of the officers of a charity by a majority of the suffrages of the Governors includes many conspicuous advantages; yet, like a multitude of other good usages, it has been subjected to great and lamentable abuses. It is well known that, previous to an election, large sums of money have been subscribed by the candidate and his friends, in order, by securing a majority of votes, to deter or defeat a less rich, or more scrupulous, competitor. This evil had acquired such a currency, and had grown to so monstrous a magnitude, that many Societies endeavoured to render such unworthy measures unavailing, by adopting wise and wholesome regulations: nevertheless, it may be feared, that the abuse is not wholly extirpated." 68.

We fear it is very far from being extirpated—and, indeed, there is little hope of freeing these and most other kinds of institutions from the influence of intrigue, party-spirit, bribery, and corruption.

The long and useful life of Mr. Hey was now drawing to a close. He had, generally speaking, enjoyed good health, and had not been afflicted with any serious bodily indisposition till the year 1805, when he suffered from a bowel-complaint, which, however, was soon subdued. In 1808, he was attacked by a painful disease, which reduced him to a state of great weakness. In December 1817, the bowel-complaint returned in an alarming degree, and Mr. H. suspected that ulceration of the colon had taken place;—although he so far recovered as to be able to go through his professional avocations, yet he never regained completely his health afterwards. He was convinced he had some organic affection that would cary him to the grave. This prediction was verified too correctly in little more than a year from the time it was uttered. Few men, at the advanced age of 82 had suffered less in their mental or corporeal faculties. His eye-sight was remarkably good, so that he could read and write without spectacles. His hand was firm, and his writing distinct. His hearing was acute—his vocal powers agreeable—and an imperfection of memory his only grievance. Yet his conceptions were clear, his judgment sound, his mode of thinking orderly, and the delivery of his ideas fluent and perspicuous. Much of the freshness and vigour which Mr. Hey possessed, is attributable, Mr. Pearson thinks, to his habits of early rising, temperance, and equanimity of mind.

After a short, and not very painful illness, Mr. Hey's earthly career was closed on the 23d of March 1819. To

To prevent repetition, we shall here give the appearances on dissection, placed (not very consistently we think) among Mr. Hey's "professional writings." It may be proper, however, first to mention, that Mr. Hey's death was deeply felt and severely lamented through the Borough of Leeds. The public papers abounded with indubitable testimonies of affectionate regret for his loss—proving that the highest estimation was attached to his superior talents, moral character, and useful virtues. We shall not notice the various effusions which appeared in the periodical prints respecting the character of Mr. Hey—since, in general, they are not to be considered as the cool convictions of the biographer. The following passage from Mr. Pearson we shall transcribe.

"His intellectual powers were of a high order. He was capable of profound investigation; was acute in discerning the differences of things; patient and diligent in his researches; possessing an ardent thirst for knowledge, combined with a sincere and sacred love of truth. Whatever object of study he deliberately took up, he pursued with resolute assiduity, until he had thoroughly made it his own, and had attained clear, comprehensive, and correct notions of it, in all its parts and relations.

"Mr. Hey was well acquainted with the works of the best practical writers in his profession; but his reading was rather select, than various and extensive, and he thought and reflected more than he read. As he was no great admirer of the theories of others, so also he was cautious and sparing in constructing any of his own; and from his observations, which were minute and accurate, he was chiefly solicitous to deduce conclusions of practical utility. As an operator he was firm, steady, collected; circumspect and deliberate in forming his determination, and not easily disconcerted by any unexpected occurrence that might present itself. Few provincial surgeons have been called to perform a greater number of the most important chirurgical operations, and perhaps none has been more successful; yet the greatness of his reputation and his acknowledged skill never seduced him into rash and hasty decisions, into presumptuous confidence, or criminal negligence: he was thoughtful, considerate, humane, and attentive to the latest period of his life. As Mr. Hey's religious principles regulated his professional conduct, he was equally sedulous in his attentions to his Infirmary patients, as to those in his private practice, and his kindness and solicitude were distributed with much impartiality to all who were under his care and superintendence. Towards his professional brethren he maintained a conduct that was upright, candid, and honourable; never withholding praise where it was due, never attempting to raise his own reputation by degrading the talents and merits of others; concealing their mistakes, where it was possible and proper, and cheerfully giving them assistance when desired. His superiority and success created jealousy and envy; and these malevolent passions, on a few occasions, broke forth in a form of opposition that was equally indecent and unchristian. The security, which his religious character gave to his adversaries against the effects of his resentment and the danger of retaliation, encouraged some base and dastardly spirits to assail his reputation and conduct with misrepresentations and slanders, which, except on one or two extraordinary occasions, he scarcely condescended to notice." 102.

With the above extract we must conclude this part of the work—professing, in common with the biographer, our admiration of Mr. Hey's character taken as a whole, but objecting to certain traits of it as bordering on superstition—a fault, however, into which the rising generation is not very likely to fall.

The length to which the biographical part of this article has run compels us to pass over unnoticed the moral, political, and theological portions of Mr. Pearson's volumes, although they contain a great deal of matter highly interesting to the medical as well as the general reader. To the religious, or as they are technically termed *serious* portions of society, these volumes will doubtless be very acceptable, as professional men have generally been considered to be much disposed to scepticism, and an unlimited believer among them is a testimony of no trifling importance.

We must now take some notice of the 3d part or small volume dedicated to the professional writings of the deceased —or rather Mr. Pearson's remarks upon them, for it is not to be expected that we shall now review works so generally in the hands of the profession. Passing over Mr. Pearson's enumeration of various papers inserted in the periodical works of the day, he next takes a short survey of Mr. Hey's principal publication, his "Observations in Surgery." In the 14th chapter, Mr. Hey treats of cancer of the penis. Twelve cases are recorded, in nine of which there was a congenital phymosis. All these were subjected to an operation, most of them to an amputation of the whole or part of the projecting portion of the penis. Ten of these recovered, and two died. The successful termination (Mr. P. remarks) of so large a portion of cases by the operation, may excite a doubt in the mind, whether all these histories ought to be regarded as examples of true cancerous affection. Mr. Pearson, whose experience in diseases of this part must be very extensive, offers the following commentary.

"1. The germination of excrescences in great quantities, and of considerable magnitude upon those parts, is not an appearance of rare occurrence, even independently of a venereal malady; and they arise more frequently where the prepuce is contracted than in other subjects, for obvious reasons. These warty substances very often discharge an ill-conditioned and fetid matter, and admit of cure by means that would exasperate a cancer.

"2. A rigidity of the internal membrane of the præputium, with a considerable thickening of the whole substance of this part attended by an offensive discharge, is not a very rare disease, especially in the middle and declining periods of life. The removal of the morbid part of the prepuce when this change of structure exists, is always an effectual cure of the malady.

"3. In all the well-marked cases of cancer of the penis which have come under the writer's observation, there has been a diseased state of some of the inguinal glands; and, notwithstanding the amputation of the penis, and the subsequent healing of the wound, the disorder of the inguinal glands has proceeded to the destruction of life. He would not assert that the amputation of the penis, in a cancerous state, has never succeeded in preserving the life of the patient; but he never had the satisfaction in his own practice, or where he has met in consultation with other surgeons, to witness so desirable an event. The inguinal glands are likewise found in a morbid state in the cancer of the labium pudendi in the female; and the disease terminates fatally. The successful issue of most of the cases recorded by Mr. Hey is the strongest evidence of the propriety of the method of treatment which he adopted; but, in amputating the penis, the writer would suggest, as the result of much experience, that instead of endeavouring to preserve the skin, as in the amputation of a limb, the operator will derive great advantage from saving as little skin as possible." 27.

In the 18th chapter of Mr. Hey's work, a case is related of an enlargement of the female mammæ from obstructed menstruation in a girl aged 14 years.* The most enlarged breast was removed, weighing upwards of eleven pounds, and after the operation, the girl began to menstruate regularly and the other breast to decrease in size.

"It is a curious fact," says Mr. Pearson, "that the female breasts sometimes enlarge after the cessation of the catamenia: and in one case where the writer was consulted by a lady of high rank, the breasts became firm, and were much larger than at any former period of her life, not excepting the time when she was bearing children. The breasts did not acquire an inconvenient size, nor was she molested afterwards by any troublesome circumstances resulting from this unexpected appearance." 28.

Mr. Hey's work received unequivocal testimonies of approbation from the most eminent surgeons in the united kingdom, and has been quoted by most writers on surgery since its appearance before the public.

---

* It is rather hazardous, we think, to conclude that any disease arises from obstructed menstruation at the age of 14 years—a period which is often, if not generally, attained, before menstruation is fully established. *Rev.*

The last of Mr. Hey's professional writings, is a paper in the 7th volume of the Medico-Chirurgical Transactions, illustrating the effects of the venereal disease on the fœtus in utero, and the modes of its communication. This being an important subject, and Mr. Pearson having dedicated a long appendix to it, we consider it proper to lay the substance of the observations of these distinguished writers fully before our readers.

Mr. Hey first describes what he considers to be the lues venerea or syphilis in the case of an infant between two and three months old. "This child had, upon its chin, a scaly eruption, extending to the angles of the lips; and its body was covered with copper-coloured spots; its voice was become stridulous, and it was extremely fretful." On enquiry, Mr. Hey was satisfied that the mother had had the venereal disease, and, indeed, that she was not, at that time, entirely free from it. Small doses of calomel produced evident amendment in the child, in the course of eight days, but the woman discontinued her attendance. "Several instances, (says Mr. Hey,) have occurred among my patients, where the mother, having been once affected by the disease, communicated it to two, three, and even four children in succession, each of them having the disease in a milder form than the preceding one, and thus, without any ground of suspicion that the mother had received a fresh infection." These cases occurred in families where Mr. Hey was the regular medical attendant, and where he assisted at the time of parturition. "This progressive communication of disease, (says Mr. H.,) to the fœtus in utero has taken place, not only where the mother has received the infection in the ordinary way, but also, where the organs of generation have remained unaffected, both in the husband and wife." He then proceeds to relate the history of a "third woman, who gained her living by drawing the breasts of women during their confinement; she became affected with ulcers at the angles of the lips, which were judged venereal;" and which Mr. Hey also "thought to be of that description." She had drawn the breasts of "a woman who was supposed to be labouring under the venereal disease; and the ulcers did not heal till they were treated as in a case of syphilis." "Several women, whose breasts were drawn by this poor woman, became infected in the manner which I shall now describe."

The case related by Mr. Hey, as illustrating the commencement and progress of the disease, is that of a Mrs. B. whose breasts were drawn twice by this woman; "in about three or four weeks afterwards she perceived a swelling of the axillary glands, and complained of soreness in her throat."

Her disease was pronounced to be venereal by a judicious surgeon, who treated it agreeably to that opinion. She continued in the use of the medicines prescribed, about five months. During this period of time she became pregnant, and "at the end of the seventh month she miscarried of a dead child. I attended her during labour, and perceived nothing amiss in the vagina, or contiguous parts." Previously to this confinement she had borne three healthy children, and had never been affected by disease in the sexual organs. In little more than a year she was delivered of a child, apparently healthy, which she suckled. "When the child was about six weeks old, an eruption, which I judged to be syphilitic, appeared upon its legs and arms." The mother and the child took mercury:—the child was in a short time freed from the eruption, but continued to take the medicine during three months. After the lapse of two months more, "two or three small ulcers appeared on the outside of the labia pudendi of the child; the mercurial course was resumed, and the ulcers were soon healed." In about five months "the nostrils became affected, the integuments of the nose were also tender; and the voice of the child grew hoarse. The mercurial course was repeated, and continued for two months. After an interval of about two months the child took the mercurial preparation again during one month; after which there was no recurrence of disease. More than two years after this period, "Mrs. B. bore another child, which was apparently healthy at its birth, and continued to be so for a few weeks. Blotches of a copper colour then appeared upon the skin, but soon disappeared upon having recourse to the mercurial medicines. After some time, (not exceeding two months,) the blotches appeared again, with a small ulcer in the labia pudendi. The child was however cured by a repetition of the treatment, and remained well." Mr. Hey proceeds thus;—"It has appeared to me from several instances, that a man may communicate the lues venerea after all symptoms of the disease have been removed, and he is judged to be in perfect health." To illustrate and confirm this opinion, the following facts are stated:—A gentleman applied to Mr. Hey under the fear of his having communicated the venereal disease to his wife. He acknowledged having had this disease before his marriage; "but from which he believed himself to have been, since that time, perfectly free. I saw no reason to doubt the truth of his declaration." "I visited his lady, and found the labia pudendi and verge of the anus beset with irregular fissures and condylomata; a discharge of puriform matter also issued from the vagina. She was advanced to the seventh month of pregnancy; but, before her delivery, at the termination of the ninth month, the diseased parts were healed, as I had pursued a mercurial course with as much vigour as seemed prudent in her condition." "The child had no other morbid appearance than an universal desquamation of the cuticle." At the end of a month "it began to have a hoarse squeaking voice, and soon exhibited a number of copper-coloured blotches upon the skin. A scaly eruption, also, appeared upon the chin; and the anus shewed an unnatural redness. I immediately commenced the mercurial course described in the first case; and the child soon got well." "Within half a year I received a letter from the lady, informing me that the child's complaint had returned." Mr. Hey sent her the prescription for the medicines which the child had previously taken, and he heard nothing more of the family. The author of this paper was aware that doubts might arise whether he had given the proper name to the disease he had described in the preceding cases. These doubts are combated by the following remarks. "When the notice of syphiloid diseases was first laid before the public, by Mr. Hunter, the chief force of his argumentation was derived from this consideration, that, although the diseases had a considerable resemblance to syphilis, yet, being cured without mercury, they were thereby proved to be of a different nature. Now, if this reasoning be allowed to have validity, we may suppose the converse of it also to be valid. If the disease in question have the usual symptoms of syphilis, and will yield to no other remedy than mercury, we may fairly conclude that it is syphilitic." He then observes, "It may justly excite surprise, that the gentleman, whose wife's case I have last related, should have remained free from disease when his wife was in the condition which I have described. I confess myself unable to account for this circumstance; I have related all that I know of the case." The concluding paragraph of this paper exhibits so honourable a testimony of the candour, liberality, and modesty of Mr. Hey, that the reader will easily excuse its being transcribed here.

"As I do not mean to enter upon an examination of the distinctions which have of late years been made betwixt genuine syphilis and syphiloid diseases, I trust I shall not be considered as having by the preceding observations expressed any doubt of the accuracy of those distinctions. I rejoice to see the candour and judgment with which every branch of medical science is now cultivated; and must ever rank myself among the pupils of those who, by their discoveries and successful labours, are daily diminishing the sufferings of mankind.*

Mr. Hey seems, in this paper, to have assumed the following positions:—

"1. That a female received the venereal infection in the angles of the lips from a woman whose breasts she had drawn, who was supposed to be in a diseased state; and that this female communicated lues venerea to several other women by drawing their breasts.

"2. That syphilis, in its secondary form, may be communicated by the mother to the fœtus in utero.

"3. That the fœtus may be contaminated when the mother has never been previously affected with lues venerea in the organs of generation, and where no apparent disease existed in the father at the time of impregnation.

"4. That the mother remaining in health, shall produce several children, each of which shall in succession become affected with syphilis a few weeks after its birth; and that the disease shall assume a milder form in each child, comparatively, until it be finally worn out.

"5. That where a man has been infected by the venereal disease during his single state, and treated for it so successfully previously to his marriage, that no symptom, primary or secondary, appeared subsequently; he shall, nevertheless, be in a capacity of communicating the venereal disease to the woman he marries, and she to her offspring.

"6. That the same man, being free from all apparent disease, and remaining so, and having been the occasion of local venereal symptoms affecting the organs of generation of his wife, shall nevertheless continue to cohabit with her without receiving any new injury." 48.

Having now presented our readers with Mr. Hey's ideas, we turn to Mr. Pearson's remarks in the Appendix.

In the first place, Mr. P. observes that, the facts connected with the history of the blind woman, are not in sufficient number to warrant the conclusion, that the sores at the angles of her lips, were venereal. The mere external characters of these sores do not authorize us to pronounce them to be syphilitic, in the absence of any history of the woman from whom she was supposed to have received the disease. The conclusion that they were venereal, seems to rest on the cure having been effected by mercury, as was those of the several women from whose breasts this female extracted milk.

"Now it is," says Mr. P. "an incontrovertible fact, that ulcers of the mouth, lips, or tongue, not syphilitic, may communicate a dis-

* Page 46.

ease, by contact, to parts destitute of the common cuticle; that all these sores will resist different modes of treatment, and finally yield to the administration of mercury; and yet the evidence that the ulcers communicating the disease did not partake of the qualities of syphilis may be indisputable. Therefore, unless it can be shown that no other kind of pus, or puriform fluid, formed upon the surface of a secreting membrane, or upon the surface of an ulcer in the substance of a part, can excite the process of ulceration when applied to a sound part which is not covered with the epidermis, the inference deduced from the simple fact of the transmission of disease from one subject to another, will not be duly substantiated." 61.

In the second place, observes Mr. P. a pregnant woman, affected with lues venerea, may contaminate the fœtus in utero, and the child born under these circumstances will be frequently found dead at birth. If living, from two to four or five weeks will commonly elapse before symptoms of syphilis appear. "It may be doubted whether an infant was ever born alive with the indubitable characters of lues venerea upon its body."

"If a woman," says Mr. P. "suffer from the secondary symptoms of syphilis during her pregnancy, the power of communicating the disease to her child cannot be influenced by the mode in which she received the infection, nor by the part to which the contagious matter was first applied. Neither will the cure of the woman by mercury, during utero-gestation, protect the fœtus from the agency of the venereal poison." 62.

Mr. Hey asserts that he has seen this extraordinary mode of conveying syphilis "in several instances." The annotator does not deny the assertion of Mr. Hey; his language, in some places, is calculated to lead to the conclusion that he has some doubt in the statement, but this is not the case. "It may be asserted, he observes, that such an order and method of transmitting syphilis from the mother to her offspring, is [are] not accordant with any known regular laws connected with the human constitution—neither has it the support of any well-established analogy." This defect of proof, however, he remarks, may be owing either to inattention, or to the very rare occurrence of the malady under so peculiar a conjunction of circumstances. It is doubtless true, as Mr. Pearson observes, that pathological science is still in a very imperfect state, and that "the history of diseases which admit of cure by the natural unassisted powers of restitution in the system, is an important desideratum which yet remains to be supplied." In support of a fact, the dissimilitude of which to the ordinary procedure of Nature Mr. Pearson is ready to acknowledge, he asserts that he has seen several instances, in which the newly married woman has had appearances bearing a strong resemblance to the primary symptoms of lues venerea, whose husband had no apparent disorder, local or constitutional, at the period of marriage nor subsequently, at the very time when his wife was suffering from the local malady.

"Every common precaution to avoid deception, and verify the statement, was used in these cases; but nothing could be collected that had a tendency to falsify the declarations of either party. It may be very properly enquired, Whether the assertor of this fact ever witnessed such an occurrence, where neither of the persons had previously suffered from venereal contamination? He replies in the negative;*—but the advantage which it may be supposed that this concession gives to an opponent, will be considerably diminished by the consideration, that the party which had formerly suffered, had no symptom of disease when he was supposed to have communicated it; that no subsequent appearance of disease occurred; and that no medicines were given to secure the person against the possibility of future inconvenience." 67.

At the same time, it is to be remembered that instances of the kind under consideration are very rare—that very few men inhabiting the large towns of Europe marry without having suffered from illicit intercourse—that a vast plurality of the women then married are consequently exposed to the hazard of receiving injury, and yet escape contamination—all these things considered, it seems warrantable to conclude that this morbid appearance only occurs in situations and conditions peculiar and not yet defined.

Mr. Hey urges the curing of these diseases by mercury as a farther evidence of this syphilitic character, adducing the authority of Mr. Hunter. Mr. Pearson's remarks on this part of the subject we shall give in his own words.

"1. That mercury is a specific remedy of syphilis may be safely admitted; yet mercury, instead of curing some diseases consequent on impure cohabitation, and especially those which resemble the chancre, will be often highly injurious to them, whether applied locally, or received into the system. Will it be asserted that these cases therefore are not venereal? But suppose that any one of these patients, and the supposition is founded on facts, after the healing of the sore without mercury, shall be infested with the secondary symptoms of lues venerea, and these symptoms shall yield to mercury; are we to conclude that the disease was originally venereal?

* "He believes that he has seen one case, in which neither of the parties had formed any sexual intercourse before marriage"

If so, then mercury may exasperate the primary symptoms of such a disease, and cure its secondary symptoms. Hence the conclusion drawn from the operation of mercury ceases, under some circumstances, to be a certain test of the presence, or absence, of syphilis.

"2. Mr. Hunter considered the gonorrhœa as one form of lues venerea; yet he was aware that the gonorrhœa often admits of a natural cure. He could not, consequently, intend to affirm, that in every venereal case, without limitation or restriction, the introduction of mercury into the system was indispensably necessary to its cure.

"3. To bear a resemblance to syphilis is an ambiguous phrase; since it may mean that some one or more of the appearances resemble those which occur in syphilis; or that the origin, progress and order of succession of the symptoms coincide exactly with those derived from the venereal poison. Now it is a remarkable and an important fact, that no one symptom derived from syphilis is peculiar to that disease; a resemblance in the evolution and mode of conjunction of the several morbid appearances is, therefore, necessary to verify and establish the identity of the morbific poisons.

"4. The evidence arising from the salutary effects of mercury in subduing the disease might be admitted as valid, if mercury cured no other malady. But as this medicine may be employed successfully in removing many cutaneous disorders, the proof fails in a most material point, and carries little or no conviction of the correctness of the inference. Indeed, when the disease 'yields to no other remedy,' and is cured by mercury; this affords a fair presumption in favour of Mr. Hey's opinion, and the evidence is strengthened, if the concurrence of symptoms resemble those of lues venerea; yet, the proof is incomplete while a discordance can be shown between the cases in question and the usual course of syphilis; and more especially if it can be shown that similar appearances occur, when nothing like a venereal taint had ever infested the mother. If the validity of 'Mr. Hunter's argumentation' be conceded; yet the converse of that is not to be admitted as equally valid. He who firmly believes that syphilis is curable by mercury only, may conclude with Mr. Hey that a given case, which has been cured without mercury, was not venereal; but since mercury may be administered with success in diseases not venereal, there is a manifest imparity between Mr. Hey's reasoning and that of Mr. Hunter.

"5. The instance of the gentleman, who, being free from any ailment, was supposed to have communicated the primary symptoms of syphilis to his wife, presents a fact not easily to be conceived, and which is still more difficult of explanation. Yet, several cases of a similar kind have fallen under the writer's observation, and what is remarkable, they have occurred more frequently where the gentleman had been infected with gonorrhœa only, and was cured previously to marriage, than when he had suffered from chancre. Some of these females had local symptoms only; in others, these were succeeded by constitutional symptoms; and in some, the malady first manifested itself by the appearance of an ulcerated throat and copper-coloured spots upon those parts of the body which are most commonly infested by venereal eruptions. But, further; symptoms bearing a great resemblance to syphilis may arise spontaneously and under circumstances where no sexual intercourse shall have occurred during many months; they may even appear after mechanical injuries received on the organs of generation, or after the application of acrid substances to those parts, and the primary sore may be succeeded by ulceration of the tonsils and copper-coloured spots on the body. These assertions are made in full confidence of the accuracy with which the observations were made. That duly verified facts like the preceding surround the subject with many difficulties, and tend to introduce a considerable measure of scepticism into the reasonings and decisions of professional men, is neither denied nor dissembled. To elucidate many conflicting facts which present themselves, no ordinary powers of discrimination may be sufficient; since the object of inquiry is sometimes enveloped in a darkness which can hardly be dispersed by talents the most brilliant; or it is involved in perplexities which refuse to be disentangled by any means short of multiplied observations, the most patient thought, well-contrived experiments, and transcendent sagacity. It may be the happy lot of some favoured mortals to keep the tenor of their way undisturbed by doubts or perplexities; and to such, he who discloses difficulties in their path, or his own, must be an object of pity, wonder, or annoyance. But doubt in medical matters is generally less dangerous than presumptuous confidence; and, if suspension of mind control rashness, it is not inimical to practical utility. The consciousness of an inability to explain, unfold, and elucidate a pathological problem is perfectly consistent with such a measure of knowledge as is quite sufficient for human utility, and which will conduct to a very safe and successful mode of treatment." 76.

With the above extract we shall take leave of this subject, which is involved in difficulties apparently insurmountable at the present moment. Time will probably cut rather than loose the Gordian knott by erasing syphilis altogether from the nosological chart of diseases. There was a period when it existed not—and why should there not arive such a period again?

A short paper closes this part of Mr. Pearson's work, containing a reply to some observations by Mr. Todd of Dublin, on a paper of the late Mr. Hey's, respecting an uncommon species of scrotal hernia. But as this paper is controversial, and accompanied by an explanatory plate, we cannot attempt to give any account of it in this place.

